# Insight into the Metabolic Profiles of Pb(II) Removing Microorganisms

**DOI:** 10.3390/molecules26134008

**Published:** 2021-06-30

**Authors:** Carla Cilliers, Evans M. N. Chirwa, Hendrik G. Brink

**Affiliations:** Department of Chemical Engineering, Faculty of Engineering, Built Environment and Information Technology, University of Pretoria, Pretoria 0002, South Africa; u14053901@tuks.co.za (C.C.); evans.chirwa@up.ac.za (E.M.N.C.)

**Keywords:** Pb(II) bio-removal, metabolic flux analysis, redox balance, bioremediation

## Abstract

The objective of the study was to gather insight into the metabolism of lead-removing microorganisms, coupled with Pb(II) removal, biomass viability and nitrate concentrations for Pb(II) bioremoval using an industrially obtained microbial consortium. The consortium used for study has proven to be highly effective at removing aqueous Pb(II) from solution. Anaerobic batch experiments were conducted with Luria-Bertani broth as rich growth medium over a period of 33 h, comparing a lower concentration of Pb(II) with a higher concentration at two different nutrient concentrations. Metabolite profiling and quantification were conducted with the aid of both liquid chromatography coupled with tandem mass spectroscopy (UPLC-HDMS) in a “non-targeted” fashion and high-performance liquid chromatography (HPLC) in a “targeted” fashion. Four main compounds were identified, and a metabolic study was conducted on each to establish their possible significance for Pb(II) bioremoval. The study investigates the first metabolic profile to date for Pb(II) bioremoval, which in turn can result in a clarified understanding for development on an industrial and microbial level.

## 1. Introduction

Lead (Pb) is a well-recognized environmental pollutant, which poses a direct threat to environmental health and well-being [1]. Pb is one of four heavy metals known to be the most damaging to human health and can affect the peripheral and central nervous systems, kidneys and blood pressure [2]. It further has the potential to accumulate in human bones with subsequent gradual release long after exposure [1]. Pb is predominantly mined in the form of galena (PbS) and cerussite (PbCO_3_) and rarely mined in pure elemental form [3]. Anthropogenic lead pollution originates mainly from leaded gasoline, coal burning, smelting, mining, waste incineration and electric battery manufacturing [3,4,5]. These non-uniform inputs of Pb result in concentrated, Pb-polluted soils and sites [4]. The World Health Organization reports that the maximum permissible limit of Pb(II) in drinking water is 0.05 ppm, which is the same concentration allowed for wastewater reported by the Environmental Protection Agency. The average concentration in reality for wastewater was, however, found to range between 200 and 500 ppm [6], which strongly motivates the aim of the current study.

Pb(II) can be removed with many conventional methods, such as chemical precipitation, cementation, coagulation, ion exchange, reverse osmosis and adsorption [6]. These methods, however, have several disadvantages, such as high costs, toxic waste materials and the safety of disposal thereof as well as ineffective removal. Using microorganisms for the safe and environmentally friendly removal of heavy metals from contaminated environments is an attractive alternative to conventional physicochemical methods [4]. Various studies have proven effective Pb(II) removal from contaminated water or soil but not the recovery of Pb(II) in elemental form. Results previously presented by this team indicated that Pb(II) bioremoval was extremely effective under anaerobic conditions using an industrially obtained microbial consortium resulting in the formation of a dark precipitate with the presence of elemental Pb [7,8,9]. The consortium was characterized in a previous study conducted by Hörstmann et al., and the key Pb(II)-resistant and -removing microorganisms were found to be *Klebsiella pneumoniae* and *Clostridium bifermentans* [9]. A summary of a few studies conducted on Pb bioremediation is presented in Table 1 below, the summary also refers to the work conducted by this team [7,9]. From these findings, it is apparent that the current study is of great interest as elemental Pb was positively identified in the precipitate as well as the ability of this consortium to remove Pb(II) concentrations of up to 2000 ppm with ease. The presence of elemental Pb is of significance as not only the removal but also the recovery of Pb is of importance to humanity as it is estimated that global Pb depletion averages at 5 Mt/year with current available Pb reserves at 85 Mt, indicating only a 17 years’ worth supply of workable raw Pb available [10,11]. 

This study aims to produce a metabolic profile for the aforementioned Pb(II)-resistant microbial consortium during bioremoval of aqueous Pb(II) from wastewater, with the use of Luria-Bertani (LB) broth as a rich growth medium. LB broth’s nutrients, metabolite content and composition were the main focus of this study, and two different concentrations of LB broth were tested. Standard LB broth contains 10 g/L tryptone, 5 g/L yeast extract and 10 g/L NaCl. Tryptone is known to be a peptone derived from the pancreatic digestion of casein, which is composed of various peptides (short chains of amino acids) [5]. Yeast extract corresponds to the soluble part of molecules released during the process of yeast autolysis or enzymatic lysis. Yeast extract contains various nutrients, and more than 50% of the yeast extract mass is made up of peptides [15]. 

The knowledge gained in the following research would be instrumental in the development of a streamlined industrial setup as it would be ideal to make use of a known compound instead of a complex mixture such as LB broth, which is expensive and difficult to optimize in terms of its exact composition. Black-box modeling and substrate analysis are currently challenging with LB broth as it contains many unknowns, and its exact composition greatly eludes the scientific community to date and is also known to vary widely [16]. The current study aimed to identify key components used in LB broth by the consortium in a “non-targeted” fashion. The knowledge acquired can give the authors insight into the inner workings of the metabolism, with the aid of energy production analysis and metabolic flux modeling. 

## 2. Results

### 2.1. Batch Reactor Results

The following Pb(II), growth and nitrate results were adapted from the first part (33 h) of the results presented by Hörstmann et al. [9]. These results are presented once more as the content forms part of the foundation for the current study, and the analyses conducted during this study were acquired from the same sample set [9].

Abiotic controls were included in a previous study by this team to verify any change being biological and not physicochemical [9]. The abiotic controls were examined over a period of 72 h, during which the Pb(II) concentration increased from 85 to 86.5 ppm, indicating a minute change in concentration (1.8%). The small increase can be attributed to accuracy variation in AAnalyst 400, which is given to be between 1% and 3% by the supplier [17]. These results confirmed that no change occurred in Pb(II) measurements without the presence of microorganisms.

**Figure 1:**
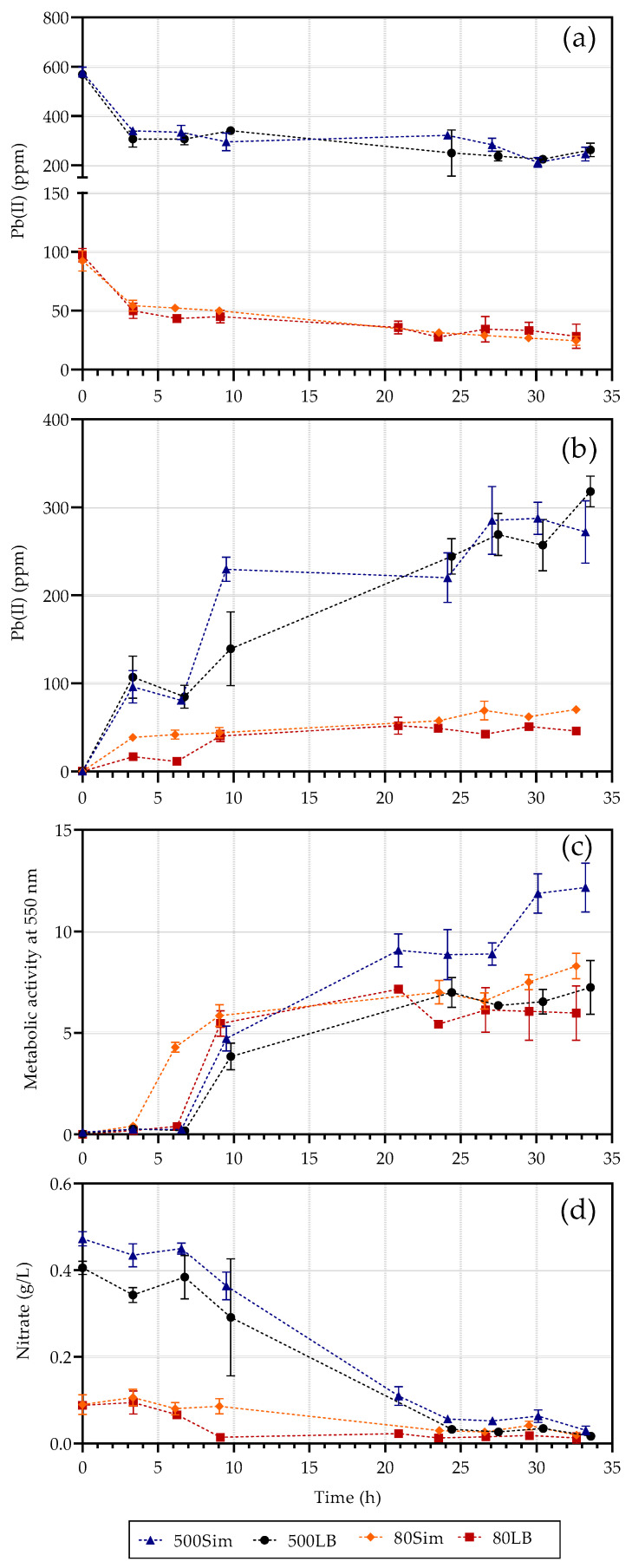
Pb(II) measurements in the supernatant (**a**) and precipitate (**b**), growth (**c**) and nitrate (**d**) measurements with time, adapted from [9].

The lead measurements in the supernatant and precipitate, growth (metabolic) and nitrate measurements are presented in Figure 1, with the error bars indicating standard deviation. Four conditions were tested, 80 ppm Pb(II) with standard LB broth (5 g/L yeast extract and 10 g/L tryptone), given as 80 LB, or simulated LB broth (10 g/L yeast extract and 20 g/L tryptone), given as 80 Sim, as opposed to 500 ppm Pb(II) with standard LB broth (500 LB) or simulated LB broth (500 Sim). This study aimed to determine the effect of the Pb(II) concentration as well as the amount of available nutrients.

Little to no difference was observed in the lead removal (Figure 1a) between the samples containing standard LB broth as opposed to simulated LB broth, indicating that nutrient concentration does not affect Pb(II) removal. The Pb(II) measurements decreased dramatically within the first three hours in all reactors without the presence of growth or nitrate usage, indicating initial lead removal independent of microbial growth. This suggests an initial biosorption mechanism at work within the first three hours with the inoculum added to the system. Substantial microbial growth was only observed after the proposed adsorption of Pb(II) onto biomass took place, indicating a possible detoxification mechanism at work. Growth was directly dependent on the nitrates available, with a drop in nitrate measurements as soon as substantial growth was observed (after approximately 6 h) (Figure 1c,d). The use of nitrates to sustain growth indicated an anaerobic respiratory mechanism present, during which nitrates can be used as an alternative electron sink in the absence of oxygen. *Klebsiella pneumoniae* and *Clostridium bifermentans* [9] are both known to respire nitrate anaerobically [18].

### 2.2. Ultra-Performance Liquid Chromatography–High-Definition Mass Spectrometry

**Figure 2:**
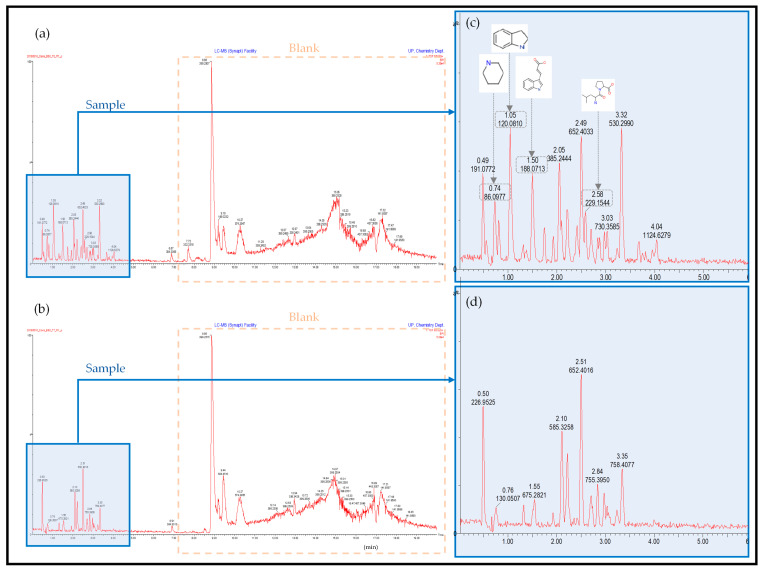
UPLC-HDMS chromatographs for 80 LB sample at 0 h (**a**), after 33 h (**b**), an enlarged image of the sample at 0 h (**c**) and 33 h (**d**).

**Figure 3:**
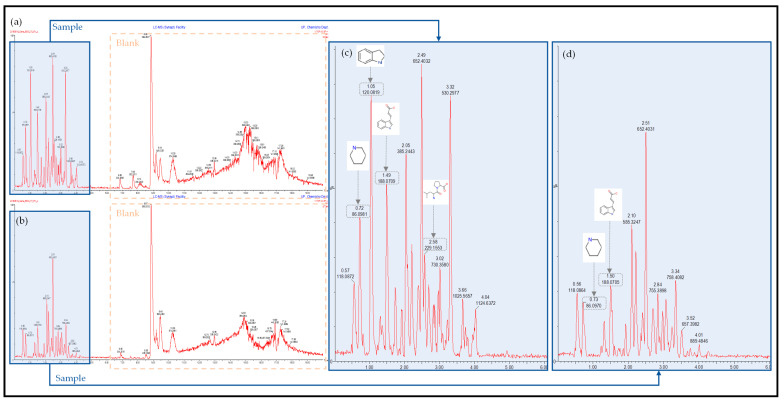
UPLC-HDMS chromatographs for 500 Sim sample at 0 h (**a**), after 33 h (**b**), an enlarged image of the sample at 0 h (**c**) and 33 h (**d**).

The UPLC-HDMS chromatograph and results for the untargeted metabolite screening are presented in Figure 2 and Figure 3 below. The screening was, however, only conducted on the 80 LB and 500 Sim samples obtained. The peaks shown in Figure 2 in the blue shaded areas are that of the sample itself at 0 h (Figure 2c) and after 33 h (Figure 2d), with the remaining peaks representing the blank used during sample preparation and analysis. This was determined by running a blank before the samples and so eliminating any irrelevant data. Simulated LB broth contained double the amount of nutrients as opposed to standard LB broth, which correlates to the larger peaks shown in the 500 Sim samples. The drop in certain peaks indicates the use of those compounds, which were selected for further study.

Four predominant peaks were identified (Table 2 below), their names, retention times, compound formulas and structures are reported. These compounds were chosen for further analyses as they were consumed the most predominantly and at higher concentrations as compared to the other compounds found in the growth medium. The aim was to determine their possible significance to the microbial metabolism during lead bioremoval. The details of each compound’s metabolic significance during anaerobic respiration are reported in the following section along with how they could have theoretically played a key part in the metabolism of Pb(II) bioremoval. All the compounds chosen were identified in both the 80 LB and 500 Sim samples and indicated with their compound structure on the spectra presented in Figure 2 and Figure 3 below.

### 2.3. High-Performance Liquid Chromatography

**Figure 4:**
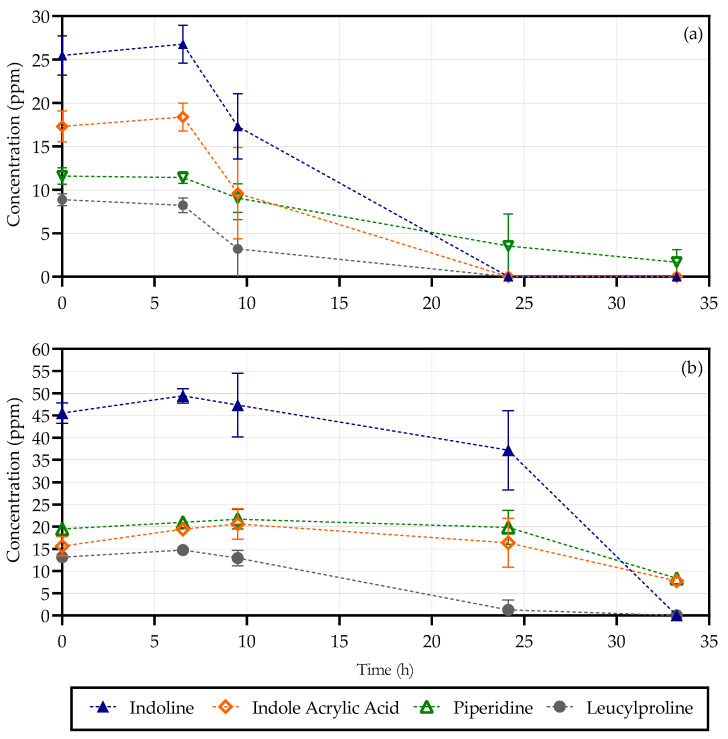
Compounds of interest for the 80 LB (**a**) and 500 Sim (**b**) samples.

High-performance liquid chromatography was used to quantitively determine the concentrations of each compound after being identified by UPLC-HDMS analysis on both the 80 LB and 500 Sim samples. The compound indoline was used as a benchmark standard to revert the data gathered from the UPLC-HDMS analysis to quantitative measurements. The UPLC-HDMS peak areas, which are directly proportional to the concentration of each compound present as they are determined on a mass basis [19], were determined and a ratio of each component to the concentration of indoline (also measured on the HPLC) was used to calculate the concentration of each. This ratio was used to determine the amount of each compound using the known concentration detected with the HPLC using an HPLC-grade standard for indoline. The results for the 80 LB and 500 Sim samples are shown in Figure 4 below.

An insignificant change in measurements was observed in the 80 LB samples (Figure 4a) for all four compounds within the first 6 h. A seemingly uniform decrease in concentrations was noted between 6 and 9 h, with only piperidine lagging the other compounds. This first uniform drop in measurements corresponds with the rapid increase in growth in the 80 LB samples, which occurred between 6 and 9 h [9]. Further utilization of the compounds was observed between 9 and 24 h. It could not, however, be concluded with certainty at what time each compound was depleted between 9 and 24 h, as the majority of compounds already measured as zero after 24 h. The only compound that was not used in its entirety was piperidine, which was not utilized at the same apparent rate as the other compounds and generally lagged behind the rest of the measurements. It should be noted that the UPLC-HDMS data were consistent with microbial growth, and no correlation was made with the decrease in Pb(II) measurements.

A significant change in compound concentrations was observed in the first 6 h in the 500 Sim samples (Figure 4b), which was similar to the observations made for the 80 LB samples. Leucylproline and indoline exhibited a slight decrease in measurements between 6 and 9 h, during the first rapid growth phase. Moderate usage of the compounds was presented between 9 and 24 h, which corresponds to the slow increase in growth observed during this time [9]. Compound depletion was only observed between 24 and 30 h, which lagged that of the 80 LB reactors, this is consistent with 500 Sim samples containing double the amount of nutrients. A dramatic decrease and depletion in indoline and leucylproline measurements were obtained between 24 and 33 h, with a slower, yet definitive, depletion rate for piperidine and indoleacrylic acid. The drop in measurements observed is consistent with the second phase of rapid growth.

The four compounds chosen amounted initially to a total concentration of 63.25 and 93.73 mg/L for 80 LB and 500 Sim, respectively. Previous findings have shown that the maximum amount of total organic carbon present in the biomass precipitates for both the 80 LB and 500 Sim samples was approximately 0.4 and 0.8 g/L, respectively [20]. These findings are thus reasonable, as only a fraction of total organic carbon was utilized. It can be concluded that only a minute amount of available substrate was used by the microorganisms to maintain growth under extreme Pb(II) conditions.

From these findings, it could be concluded with confidence that certain nutrients are used in a quicker fashion than others, and further investigation into their significance in the overall metabolism needs to be carried out.

### 2.4. X-ray Photoelectron Spectroscopy

XPS results were acquired with to aim to shed light on the precipitate identity and give more insight into the microbial metabolism. The XPS results for the 80 LB samples as reported in a previous project [21] are compared to new data for the 500 Sim samples after 6 days of experimentation in Table 3 below.

A significant amount of PbS was observed in both 80 LB and 500 Sim samples. Sulfur was not added during the experiments, suggesting the release of sulfur from the catabolism of sulfur-containing amino acids (cysteine and methionine) present in LB broth (yeast extract and tryptone). The remainder of each precipitate consisted of PbO and Pb^0^. The findings strongly indicate that PbO is the result of unavoidable oxidation of Pb^0^. The samples were prepared anaerobically but were difficult to maintain, as the samples were not centrifuged and washed anaerobically, and oxygen ingress was highly probable. The formation of PbO from Pb^0^ rests on the knowledge that only PbS, PbO and Pb^0^ are present in all the samples. The initial oxidation of Pb(II) to PbO, before sample preparation could not be responsible for the formation of PbO as no precipitate was formed in any abiotic controls and the Pb measurements remained constant. The other option could have been the oxidation of PbS at intermediate temperatures, which would have produced the partial oxidation of PbS to PbSO_4_ and PbO, as opposed to the complete oxidation of PbS to PbO, which could only occur at temperatures exceeding 800 °C [22]. From these findings, it can be deduced that the PbO was not a result of PbS or the initial chemical oxidation of Pb(II). It can be concluded that the PbO was probably due to direct microbial precipitation or the oxidation of Pb^0^ during sample preparation for XPS analysis. The atmospheric reaction of PbO from Pb^0^ has an overall spontaneous redox reaction of 2 Pb + O_2_ → 2 PbO with ΔG < 0. Oxygen would, however, be required in the current Pb removal system, most likely from H_2_O, followed by the release of H^+^ and so resulting in a decrease in pH. The pH was unchanged before and after anaerobic experimentation, ranging between 5.5 and 6.5, indicating that this reaction would not be the likely culprit for the formation of PbO. It can, thus, be concluded that the precipitate initially only consisted of PbS and Pb^0^ [21].

## 3. Discussion

### 3.1. Metabolites

Research was conducted on each of the metabolites identified. The following section reports results for each compound as well as the proposed possible metabolic flux pathways for energy generation. The pathways were presented using literature and considering the likelihood of anaerobic microorganisms (with the tendency to denitrify) using the pathway and mechanism. The pathways suggested in this part of the study were adapted from research conducted on anaerobic respiratory microorganisms. The products produced from one molecule of each compound are of interest as this could be used for energy production analyses. The aim was to give more insight into the preferential degradation of these compounds observed in the UPLC-HDMS and HPLC results and to elucidate the motivation of the microbial consortium for energy production.

#### 3.1.1. Piperidine

**Figure 5:**
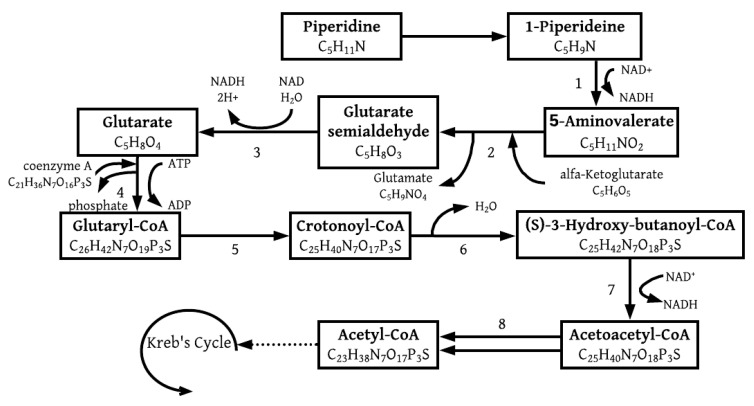
Catabolic pathways for piperidine degradation coupled with the lysine metabolism, enzyme activities are 1: 1-piperideine dehydrogenase; 2: 5-aminovalerate aminotransferase; 3: glutaric semialdehyde dehydrogenase; 4: glutaryl-CoA synthetase; 5: glutaryl-CoA dehydrogenase; 6: enoyl-CoA hydratase; 7: 3-hydroxyacyl-CoA dehydrogenase; 8: acetyl-CoA C-acetyltransferase [26,27].

Piperidine, a heterocyclic amine, lysine-derived alkaloid and a metabolite of cadaverine plays a key role in the process of lysine degradation [23]. The degradation of heterocyclic compounds has been of great interest of late as it is popular for industrial activities, such as the manufacturing of rubber additives, drugs and herbicides. This high demand for industrial purposes led to the release of these chemicals into the environment [24]. The degradation of piperidine has been proven to be effective for denitrifying proteobacteria such as *Thauera*, *Castellaniella*, *Rhizobium*, or *Paracoccus* [24], where anaerobic degradation of piperidine was coupled with nitrate reduction. This is of interest as it is shown in this study that nitrate reduction was not only present in the system but instrumental in the efforts to sustain its metabolism and Pb(II) removal. Lysine is an essential amino acid for many living organisms [25] and can be degraded through various metabolic pathways. Two prominent pathways were identified for lysine degradation, one producing succinate and the other acetyl-CoA. The route producing succinate would be impossible, as it requires molecular oxygen within one of its steps. The likely pathway for lysine degradation is presented in Figure 5 below. The pathway branches off from glutarate to glutaryl-CoA as an intermediate step.

Regarding Figure 5, acetoacetyl is given as C_4_H_4_O_2_ and acetyl as C_2_H_2_O, which indicates that acetoacetyl-CoA produces two molecules of acetyl-CoA. This also means that two acetyl-CoA molecules were produced from one molecule of piperidine, which is of importance when analyzing the total ATP production after completion of both the TCA cycle and the electron transport chain.

#### 3.1.2. Indoline

**Figure 6:**
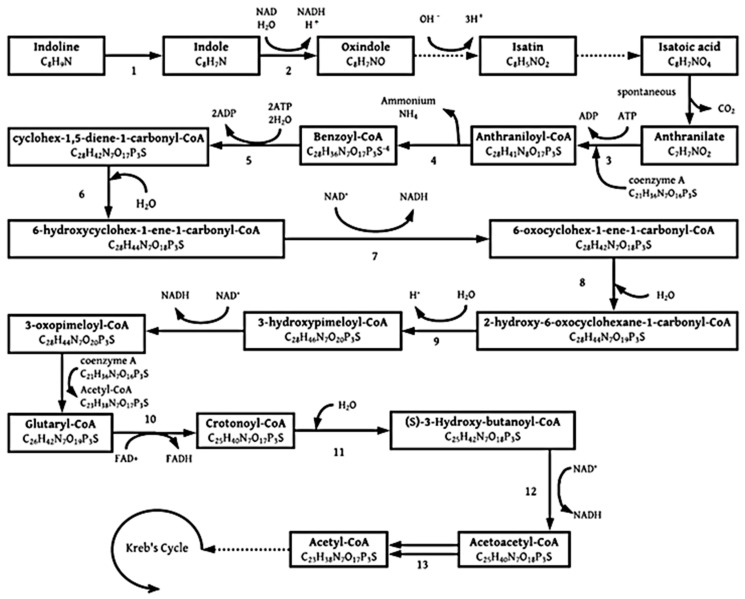
Catabolic pathway for anaerobic indoline degradation, enzyme activities are 1, dehydrogenases; 2, monooxygenase [33]; 3, aminobenzoate-CoA ligase; 4, 2-aminobenzoyl-CoA reductase; 5, benzoyl-CoA reductase; 6, cyclohexa-1,5-diene-1 carboxyl-CoA hydratase; 7, 6-hydroxycyclohex-1-ene-1-carbonyl-CoA dehydrogenase; 8, 6-oxocyclohex-1-ene-1-carbonyl-CoA hydrolase; 9, 6-oxocyclohex-1-ene-1-carbonyl-CoA hydrolase; 10, glutaryl-CoA dehydrogenase; 11, enoyl-CoA hydratase; 12, 3-hydroxyacyl-CoA dehydrogenase; 13, acetyl-CoA C-acetyltransferase [26,27,28,29,30,31,32,33].

Indoline is a 2,3-dihydroindole, which is a saturated structural analog of indole. It can be catalyzed to indole with dehydrogenases, a dehydrogenation enzyme [28]. Indole is a N-heterocyclic aromatic pollutant regularly found in industrial and agricultural wastewater. Indole and its derivatives are metabolized from tryptophan by tryptophanase by many bacterial species [29]. Denitrifying bacteria have been found to be effective at degrading indole. The proposed pathway under anaerobic conditions is presented in Figure 6 below. The pathway starts with indoline, which is dehydrated to indole, followed by oxindole, isatin, isatoic acid, and then anthranilate [20,24]. Various pathways could be taken after the production of anthranilate. Anaerobic degradation with denitrifying bacteria of indole possesses many similarities to aerobic degradation of indole [30]. The anaerobic degradation of anthranilate, however, looks considerably different from that of aerobic degradation. Aerobic organisms use the available oxygen and oxygenases to add hydroxyl groups and cleave the aromatic ring present. The route followed for anaerobic indole degradation is presented in Figure 6 below; it was adapted from various literature [26,27,28,29,30,31,32]. 

From Figure 6 and keeping in mind that two acetyl-CoA molecules are formed from acetoacetyl-CoA and one just before the formation of glutaryl-CoA, it is established that three acetyl-CoA molecules are produced from one indoline molecule.

#### 3.1.3. Indoleacrylic Acid

Indoleacrylic acid, also known as indoleacrylate, degrades enzymatically to indole. The steps followed to produce indole are shown below with the remainder of the proposed pathway as described in Figure 6 above. Indole acrylic acid degrades to form tryptophan and then finally indole [34]. Indoleacrylic acid plays an important part in microbial physiology, e.g., acting as a signaling molecule as well as aiding the organism to resist multiple stresses, which include salinity and UV, and most importantly for activation of the TCA cycle when overproduced [35].

**Figure 7:**
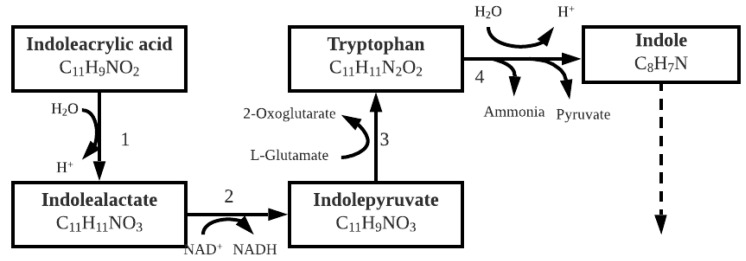
Catabolic pathway for indoleacrylic acid degradation, enzyme activities are 1: indolelactate dehydratase; 2: indolelactate dehydrogenase; 3: aromatic amino acid aminotransferase; 4: tryptophanase [34].

From Figure 7, it is clear that one pyruvate is produced from one molecule of indoleacrylic acid as well as one indoline, from which it was found earlier that that acetyl-CoA molecules are produced.

#### 3.1.4. Leucylproline

**Figure 8:**
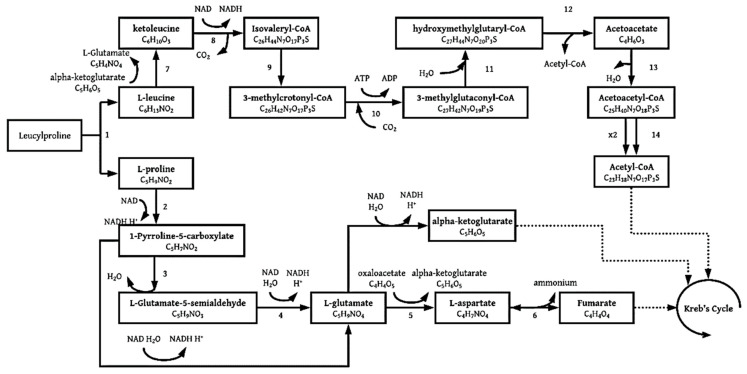
Catabolic pathway for leucylproline degradation, enzyme activities are 1: peptidase enzyme; 2: PRODH proline dehydrogenase [39]; 3: non-enzymatic; 4: P5 CDH L-glutamate γ-semialdehyde dehydrogenase [39]; 5: aspartate aminotransferase [40]; 6: aspartate ammonia-lyase [40]; 7: 2-oxoglutarate aminotransferase [41]; 8: 4-methyl-2-oxopentanoate dehydrogenase; 9: isovaleryl-CoA:FAD oxidoreductase, isovaleryl-CoA dehydrogenase; 10: 3-methylcrotonyl-CoA carboxylase; 11: 3-methylglutaconyl-CoA hydratase; 12: hydroxymethylglutaryl-CoA lyase; 13: 3-oxoacid CoA-transferase; 14: acetyl-CoA C-acetyltransferase [42].

Leucylproline belongs to a group of organic compounds known as dipeptides, specifically from the X-pro type [36]. Dipeptides contain exactly two alpha-amino acids (leucine and proline [37]), which are joined at the peptide bond [38]. Leucylproline can be hydrolyzed microbially to produce L-leucine and L-proline [35,36] with a specific peptidase enzyme [36]. The L-proline and L-leucine are then degraded separately, and their degradation pathways are presented in Figure 8 below.

L-proline can be microbially degraded to L-glutamate [39], which is then followed by the formation of fumarate through various steps proposed in Figure 8 below [40].

It was gathered from Figure 8 that three molecules of acetyl-CoA are formed from one molecule of L-leucine. Various pathways exist for proline degradation, it was, thus, chosen to continue with the path that produced the most energy, which was from proline to L-glutamate-5-semialdehyde, through glutamate directly to alpha-ketoglutarate.

### 3.2. Energy Production Analysis

The amount of energy produced from one molecule of each metabolite was calculated in the following part of the study. An estimation was made on the amount of NADH and FADH produced using one mol of each compound using 80 LB samples only. The calculations were based on the entry point into the TCA cycle as well as the initial amount of NADH and FADH produced. The results are presented in Table 4 below. The table indicates the initial NADH and FADH that are produced before entering the TCA cycle as well as during the TCA cycle. The net amount NADH and FADH produced was calculated using the concentrations calculated for 80 LB only from the above as well as the molar mass of each component (Figure 4).

It was confirmed that indoleacrylic acid, leucylproline and indoline were used preferentially in the 80 LB samples compared to piperidine. This directly corresponds to the amount of total NADH generated during the use of one piperidine molecule. When observing the 500 Sim samples, it was noted that indoline and leucylproline were consumed at a higher apparent rate within the first phase of rapid growth (9 to 24 h). Between 24 and 33 h, it was observed that indoline and leucylproline were depleted with piperidine and indoleacrylic acid reserves available at the end of the experiments, during which it was concluded that a second stage of growth was initiated due to substrate availability. These findings also correspond to the NADH calculations from above, except for indoleacrylic acid in the 500 Sim samples. The reason for this exception can possibly be attributed to the high abundance of overall nutrients available in the 500 Sim samples as compared to the 80 LB samples.

### 3.3. XPS Coupled with Metabolite Profiling

The results from XPS analysis are further described in the following with relation to the amount of Pb(II) removed from the samples as described in 2019 [21] and 2020 [9] by this team. This section aims to determine whether there are sufficient nutrients present to interact with the amount of Pb found in the pellet (precipitate) as well as the amount needed for anaerobic denitrification.

From a combination of these results, it was found that 67.9 ppm Pb(II) was present in the pellets of the 80 ppm (80 LB) samples and 254.7 ppm in the 500 ppm (500 Sim) samples after 6 days [21] with an XPS ratio of 0.46:0.54 and 0.41:0.59 to (PbO and Pb^0^):(PbS), respectively.

The pellet Pb measurement after 33 h for 80 LB was found to be 45.83 ppm (Figure 1b). The focus of this section will be on the 80 LB samples and not the 500 Sim samples assuming that the 80 LB samples’ PbS content has stabilized, and the 500 Sim has yet to do so. The two balanced equations for both nitrate and Pb are presented in Equations (1) and (2) below.

(1) $${\mathrm{NO}}_{3}^{-}+4\;\mathrm{NADH}+6H^{+}\to {\mathrm{NH}}_{4}^{+}+4{\mathrm{NAD}}^{+}+2H_{2}O$$

(2) $${\mathrm{Pb}}^{2+}+\mathrm{NADH}\to {\mathrm{Pb}}^{0}+{\mathrm{NAD}}^{+}+H^{+}$$

The average molecular weight was calculated for PbS, Pb and PbO, which was in turn used to determine the molecular concentration of Pb in the pellet, producing a value of 0.29 mmol/L. The XPS fractions and individual molecular weights were used to determine the concentration of each Pb species as indicated in Table 5 below.

From these results, it was calculated that 0.035 mmol/L of NADH is required to successfully reduce Pb(II) to elemental Pb.

The initial amount of nitrate present as reported in 2020 [9], was given as 0.9 g/L, which translated to a molecular concentration of 1.5 mmol/L. The amount of NADH required to reduce nitrate was, thus, calculated to be 5.8 mmol/L NADH. The final theoretical amount of NADH required by this system was then calculated by adding the amount of NADH needed for Pb(II) and nitrates to produce a value of 5.84 mmol/L.

The net amount of NADH produced was found to be 10.4 mmol/L (Table 4). Indicating that the amount of NADH produced was sufficient to satisfy the amount needed for nitrate and lead reduction. The surplus of energy produced may be explained by the NADH needed to satisfy biomass redox requirements.

## 4. Materials and Methods

### 4.1. Materials

The batch reactors were set up anaerobically in 100 mL serum bottles, each spiked with a Pb(II) stock solution made with Pb(NO_3_)_2_ (Merck, Kenilworth, NJ, USA). A rich growth medium was used, standard Miller Luria-Bertani (LB) broth (Sigma Aldrich, St. Louis, MO, USA) as well as simulated LB broth, which contained double the amount of nutrients and a decreased amount of NaCl to promote microbial growth. Nitrate measurements were continuously measured photometrically using nitrate-testing kits (Merck, Darmstadt, Germany) and a Spectroquant Nova 600 (Merck, Darmstadt, Germany). Growth was quantified using 3-(4,5-dimethylthiazol-2-yl)-2,5-diphenyl tetrazolium bromide (MTT) coupled with an organic solvent dimethyl sulfoxide (DMSO) (Sigma Aldrich, St. Louis, MO, USA) and measured at 550 nm.

An indoline (HPLC grade, ReagentPlus^®^, 99%) standard was used as a benchmark for HPLC analysis (Sigma-Aldrich, Bryanston, Sandton, South Africa). The standard, as well as samples, was dissolved in methanol (HPLC grade, gradient grade, ≥99.9%, Sigma-Aldrich). The samples and standard were made directly before analysis on the HPLC. TFA (trifluoroacetic acid, HPLC grade, ≥99.0%, Sigma-Aldrich) was used with Ultrapure water as solvent B and methanol solvent C for the HPLC.

### 4.2. Microbial Culture

The locally obtained lead-resistant microbial consortium was collected from a borehole in Gauteng, South Africa, at an automotive battery recycling plant. The original inoculum was cultured by adding 1 g of lead-contaminated soil to a 100 mL anaerobic (by purging the reactor with nitrogen gas for 3 min) serum bottle containing LB and spiked with 80 ppm Pb(II). The batch reactors were incubated overnight at 32 °C and 120 rpm. The cultures were subsequently cryogenically stored at 77 °C with glycerol at a 20% *v*/*v* ratio. Precultures were prepared from the cryogenically stored culture, by inoculating an anaerobic batch reactor with one loop of inoculum at both 80 and 500 ppm Pb(II) in standard LB broth. The batch reactors were incubated at 35 °C and 120 rpm. These precultures were stored cryogenically in the same manner as the inoculum and used directly after thawing for each experiment.

### 4.3. Experimental

The growth medium (standard or simulated LB broth) and Pb(II) stock solution was autoclaved separately and cooled to room temperature. The Pb(II) stock solution was added in 100 mL serum bottles to the growth medium in a strictly sterile environment. The serum bottles were inoculated with 0.2 mL of stored and thawed preculture, purged for 3 min with nitrogen gas and sealed off to maintain anaerobiosis. The reactors were incubated at 35 °C at 120 rpm for the entire period of experimentation. Abiotic controls were conducted in parallel to the experiment for 72 h to confirm all changes as being microbial as opposed to physicochemical.

### 4.4. Sampling

Samples were taken over 33 h every 3 h at 3, 6, 9, 24, 27, 30 and finally at 33 h. The sealed anaerobic batch reactors were roughly shaken and pierced with a hypodermic needle and sterile syringe. The samples used for metabolic activity were measured immediately and the rest stored for later analyses.

### 4.5. Batch Analyses

#### 4.5.1. Pb(II) Measurements

Residual aqueous Pb(II) measurements were quantified by measuring the diluted sample supernatants on an atomic absorption spectrometer (Perkin Elmer AAnalyst 400, Waltham, MS, USA). Measurements were obtained with a Pb Lumina hollow cathode lamp, a wavelength of 283.32 nm, an atomic adsorption slit of 2.7/1.05 mm, linear to 10 mg/L, oxidant (air) flow of 10.0 L/min and acetylene flow of 2.5 L/min. The flame slit was 1.8/0.6 mm, flame emission wavelength was 405.78 nm, N_2_O oxidant at 6.0 L/min and acetylene flow of 7.5 L/min.

#### 4.5.2. Metabolic Activity

Growth (metabolic activity) measurements were recorded, at the time of sampling, photometrically at 550 nm using MTT dissolved in organic solvent DMSO [43]. Two sets of measurements were obtained and deducted from each other to avoid any background interference, namely with biomass and without. The samples without biomass were filtered with 25 mm nylon syringe filters with 0.45 m pores (Anatech, Randburg, South Africa). MTT was added to the samples (with or without biomass) after dilution and incubated for 60 min at 35 °C. The samples were dissolved in DMSO after incubation and their absorbances were finally measured at 550 nm.

#### 4.5.3. Nitrates

Nitrate measurements were recorded after experimentation using nitrate-testing kits, where nitrate ions react with a form of benzoic acid and sulfuric acid to produce a red nitro solution which was, in turn, measured photometrically with a Super Nova 600 (Merck, Darmstadt, Germany).

#### 4.5.4. UPLC-HDMS

The LC-MS data was acquired through the LC-MS Synapt facility of the University of Pretoria’s Department of Chemistry. Ultrapure LC water (Romil-UpS™, Microsep, Johannesburg, South Africa) and formic acid (99+% purity) (Fluka^®^ Analytical, Sigma-Aldrich, Sandton, South Africa). A Kinetex^®^ 1.7 μm EVO C18 100 Å (2.1 mm ID × 100 mm length) column was used during analysis (Phenomenex, California, USA). The analysis was performed with Waters^®^ Aquity UPLC-qTOF Synapt G2 high-definition mass spectrometer (UPLC-HDMS) (Waters, MA, USA). The system consisted of a Waters Acquity UPLC^®^ connected to a quadrupole-time-of-flight instrument [44].

The supernatants (unfiltered) of the samples were diluted to a range of 1–10 ng/uL. The MS system was operated in positive ion mode and mass spectra were acquired. The exact molecular mass data from redundant *m/z* peaks were used to help confirm the compound molecular masses. The MassLynx^TM^ (version 4.1) application (Waters, MA, USA) was used to accommodate data collection. Peak areas were quantified by selecting each target compound’s molecular mass ions (M + H)^+^. Untargeted identification was achieved by matching the acquired retention times, mass and MS/MS fragmentation to certified standards. This comparison was achieved with the online verification of known compounds on ChemSpider (http://www.chemspider.com/ accessed 1 July 2019).

#### 4.5.5. HPLC Conditions and Specifications

The specific compounds identified during the untargeted metabolic profiling (UPLC-HDMS) were analyzed further with the aid of a high-performance liquid chromatography system (HPLC name). Various studies have shown that reverse HPLC is an effective method for peptide and metabolite analysis and quantification. The method developed for this study was founded on a method described for analysis of indole compounds in sugar cane juice [14]. The study mentions using 0.1% formic acid with water as well as methanol as mobile phase. The measurements were conducted at a wavelength of 280 nm and isocratically 20% methanol and 80% formic acid at 30 °C. It was, however, thought best to use TFA as various studies have shown that it is more effective than formic acid as it has a lower UV absorption and high peak capacity as opposed to formic acid [45].

A Waters 2695 Separations module was used coupled with a 2489 UV/Vis detector that was set at 280 nm alongside a Waters PAH C_18_ 5 µm 4.6 mm × 250 mm column (Waters, Milford, MA, USA).

The system was run isocratically with methanol (solvent C), coupled with 0.1% TFA (solvent B) at a 20:80 ratio, respectively. The flow rate was kept constant at 0.6 mL/min, with an ejection size of 5 µL and 30 °C. The system was washed between each sample to reduce carry-over.

#### 4.5.6. XPS Sample Preparation and Analysis

X-Ray photoelectron spectroscopy (Thermo ESCAlab 250 Xi, Waltham, MA, USA) was performed on the 80 LB and 500 Sim precipitates, the samples retained their original form and only a small amount of precipitate was required.

The samples were centrifuged at 9000 rpm at 20 for 10 min, decanted and then washed six times. The washed precipitates were then dried anaerobically in a desiccator overnight with silica crystals, an anaerobic indicator (Oxoid, Thermo Scientific, Basingstoke, Hampshire, UK) and an AneroGenTM sachet (Oxoid, Thermo Scientific, Basingstoke, Hampshire, UK).

## 5. Conclusions

The study shared insights into the metabolism of a Pb(II)-resistant microbial consortium with the main focus on its key microorganisms previously identified as *Klebsiella pneumoniae* and *Clostridium bifermentans*. Four main compounds were identified using UPLC-HDMS, and further study was conducted into their possible metabolic importance to Pb(II) removal. The findings were compared to previous Pb(II) removal, growth and nitrate measurements. These four components were identified as piperidine, indoline, indoleacrylic acid and leucylproline. Their concentrations were quantified using HPLC measurements in conjunction with the data obtained from UPLC-HDMS analyses, and it was found that these compounds were preferentially consumed over time. The possible metabolic degradation pathways were reported from literature, and total NADH and FADH productions were calculated. The calculated energy productions were found to be 4.87, 0.68, 3.36 and 1.49 mmol/L for indoleacrylic acid, leucylproline, indoline and piperidine, respectively. It was found that indoleacrylic acid, leucylproline and indoline was used preferentially to piperidine in samples containing 80 ppm Pb(II) and standard LB broth. For samples containing 500 ppm Pb(II) and simulated LB broth, it was found that indoline and leucylproline were depleted before piperidine and indoleacrylic acid. From these findings, it could overall be concluded that the compounds that were theoretically able to produce higher amounts of overall NADH and FADH were consumed preferentially.

It was also calculated that the amount of theoretical NADH produced by the four compounds was 10.4 mmol/L, which was more than enough both for the reduction of Pb(II) and for nitrate depletion (only 5.84 mmol/L), with the remainder of the NADH available likely dedicated to cell growth and maintenance. It is recommended that further studies should be conducted into the pathways currently proposed to obtain more accurate and definitive results.

These findings shed light for the first time on the possible metabolic behavior of Pb(II)-resistant microorganisms, that are capable of removing Pb(II) from wastewater in elemental form. The results could, in turn, support further research into its possible metabolic pathways and the biochemistry needed for the development of a continuous setup on an industrial scale. The results put forward will be able to aid in the optimization and refining of Pb(II) bioremoval by simplifying the ideal substrate needed for Pb(II) removal.

## Figures and Tables

**Table 1:** Pb bioremediation examples compared to the current study.

Microorganism Type and Name	Pb Contamination Type	Mechanism	Maximum Pb Concentration	Removal (%)	Precipitate Identity	Reference
Bacteria *Kocuria flava*	Soil	Calcite precipitation	85.4 mg/kg	83.4	Various Pb calcite species	[4]
Microalgae *Nannochloropsis oculata*	Water	Pb absorption	1.3 ppm	55	Not applicable	[12]
Microbial consortium	Water	Biosorption	50 ppm	57	Not mentioned	[13]
Bacteria *Bacillus* sp.	Water	Biosorption	518 ppm	37	Pb_5_(PO_4_)_3_ OH	[14]
Microbial consortium (mainly *Klebsiella pneumoniae* and *Clostridium bifermentans*)	Water	Biosorption coupled with bio-precipitation	2000 ppm	99	PbS, PbO, Pb^0^	[7,9]

**Table 2:** Detected compounds from untargeted metabolite screening with the UPLC-HDMS.

Compound (Mass Spectrometry Similarity ≥90%)	Mass (g/mol)	Retention Time (min)	Formula	Structure
Piperidine	85.15	0.72–0.74	C_5_ H_11_ N	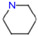
Indoline	119.1	1.05–1.054	C_8_ H_9_ N	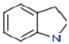
Indoleacrylic Acid	187.1	1.49–1.5	C_11_ H_9_ NO_2_	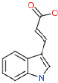
Leucylproline	228.2	2.58	C_11_ H_20_ N_2_ O_3_	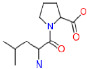

**Table 3:** XPS results comparing the relative presence of the three Pb species found for the two concentrations, respectively.

Dataset	PbS (%)	PbO (%)	Pb^0^ (%)
80 LB [21]	54	38	8
500 Sim	59	41	0

**Table 4:** Overall NADH production for the identified compounds.

Compound	Product	NADH_i_	FADH_i_	NADH_TCA_	FADH_TCA_	Net NADH and FADH (mmol/L)
Piperidine	2 × Acetyl-CoA	4	0	3 × 2 = 6	1 × 2 = 2	1.49
Indoline	3 × Acetyl-CoA	5	0	3 × 3 = 9	1 × 3 = 3	3.36
Leucylproline	3 × Acetyl-CoA + Fumarate	4	0	3 × 3 + 2 = 11	1 × 3 + 1 = 4	0.68
Indoleacrylic acid	4 × Acetyl-CoA	7	0	3 × 3 + 3 = 12	1 × 4 = 4	4.87
Total						10.4

**Table 5:** Concentration of Pb species after 33 h and 6 days (144 h).

PB SPECIES	Concentration after 33 h (ppm)	Concentration after 144 h (ppm) [9]
PBS	38.04	38.04
PBO	7.79	24.98
PB^0^		4.88
TOTAL	45.83	67.9

## Data Availability

The data presented in this study are openly available in the University of Pretoria Research Data Repository at 10.25403/UPresearchdata.14873205.

## Sample Availability

The cultures used in this study are available from the authors.
